# Causal associations between *Helicobacter pylori* infection and pregnancy and neonatal outcomes: a two-sample Mendelian randomization study

**DOI:** 10.3389/fcimb.2024.1343499

**Published:** 2024-03-14

**Authors:** Jialyu Huang, Yuxin Liu, Dingfei Xu, Mengyi Chen, Qiqi Xie, Jia Chen, Leizhen Xia, Lamei Yu, Qiongfang Wu, Zengming Li, Jiawei Wang, Lifeng Tian

**Affiliations:** ^1^Center for Reproductive Medicine, Jiangxi Maternal and Child Health Hospital, National Clinical Research Center for Obstetrics and Gynecology, Nanchang Medical College, Nanchang, China; ^2^Department of Clinical Medicine, School of Queen Mary, Nanchang University, Nanchang, China; ^3^Department of Obstetrics, Jiangxi Maternal and Child Health Hospital, National Clinical Research Center for Obstetrics and Gynecology, Nanchang Medical College, Nanchang, China; ^4^Key Laboratory of Women’s Reproductive Health of Jiangxi Province, Jiangxi Maternal and Child Health Hospital, National Clinical Research Center for Obstetrics and Gynecology, Nanchang Medical College, Nanchang, China; ^5^Reproductive and Genetic Hospital, The First Affiliated Hospital of USTC, Division of Life Sciences and Medicine, University of Science and Technology of China, Hefei, China

**Keywords:** *Helicobacter pylori*, pregnancy, Mendelian randomization, preeclampsia, premature rupture of membranes

## Abstract

**Background:**

Observational studies have reported that *Helicobacter pylori* (*H. pylori*) infection is associated with a series of pregnancy and neonatal outcomes. However, the results have been inconsistent, and the causal effect is unknown.

**Methods:**

A two-sample Mendelian randomization (MR) study was performed using summary-level statistics for anti-*H. pylori* IgG levels from the Avon Longitudinal Study of Parents and Children Cohort. Outcome data for pregnancy (miscarriage, preeclampsia-eclampsia, gestational diabetes mellitus, placental abruption, premature rupture of membranes, postpartum hemorrhage) and neonates (birthweight, gestational age, and preterm birth) were sourced from genome-wide association meta-analysis as well as the FinnGen and Early Growth Genetics Consortium. Causal estimates were calculated by five methods including inverse variance weighted (IVW). The heterogeneity of instrumental variables was quantified by Cochran’s Q test, while sensitivity analyses were performed via MR-Egger, MR-PRESSO, and leave-one-out tests.

**Results:**

IVW estimates suggested that genetically predicted anti-*H. pylori* IgG levels were significantly associated with increased risks of preeclampsia-eclampsia (odds ratio [OR] = 1.12, 95% confidence interval [CI] 1.01–1.24, *P* = 0.026) and premature rupture of membranes (OR = 1.17, 95% CI 1.05–1.30, *P* = 0.004). Similar results were obtained for preeclampsia-eclampsia from the MR-Egger method (OR = 1.32, 95% CI 1.06–1.64, *P* = 0.027) and for premature rupture of membranes from the weighted median method (OR = 1.22, 95% CI 1.06–1.41, *P* = 0.006). No significant causal effects were found for other outcomes. There was no obvious heterogeneity and horizontal pleiotropy across the MR analysis.

**Conclusion:**

Our two-sample MR study demonstrated a causal relationship of *H. pylori* infection with preeclampsia-eclampsia and premature rupture of membranes. The findings confirm the epidemiological evidence on the adverse impact of *H. pylori* in pregnancy. Further studies are needed to elucidate the pathophysiological mechanisms and assess the effectiveness of pre-pregnancy screening and preventive eradication.

## Introduction

*Helicobacter pylori* (*H. pylori*) is a gram-negative bacterium with urease, catalase, and oxidase activity that colonizes the human stomach (Zamani et al., 2018). It is one of the most common pathogens in the world, infecting more than half of the whole population (Kamboj et al., 2017). Therefore, the health impact of *H. pylori* infection, such as peptic ulcer disease, chronic gastritis, gastric adenocarcinoma, and gastric cancer, is crucial for public health (Kusters et al., 2006; McColl, 2010). Among pregnant women, the prevalence of *H. pylori* infection remains high in many countries (Baingana et al., 2014; Mubarak et al., 2014) and it has been suggested that increased susceptibility to *H. pylori* infection may be due to pregnancy itself (Lanciers et al., 1999).

A systematic review of studies published up to November 17th, 2018, exploring associations of *H. pylori* infection with pregnancy and neonatal complications, showed significantly increased risks of preeclampsia, gestational diabetes mellitus, spontaneous miscarriage, and low birthweight (Zhan et al., 2019). Consistently, some other studies also reported an adverse effect in certain outcomes (Wanyama et al., 2016; den Hollander et al., 2017; Li et al., 2020; Tang et al., 2021). In particular, for adverse pregnancy outcomes, *H. pylori* infection is associated with gestational diabetes mellitus (Li et al., 2020; Tang et al., 2021) and preeclampsia (Tang et al., 2021), while for adverse neonatal outcomes, *H. pylori* infection is associated with low birthweight (den Hollander et al., 2017; Wanyama et al., 2016) and small for gestational age (den Hollander et al., 2017). However, most single studies examined only one or few outcomes without a comprehensive evaluation. In addition, these observational studies were vulnerable to residual confounding, and variations in confounder control could cause heterogeneity between studies, thereby leading to controversial results. Thus, reexamining the impact of infections on a range of pregnancy and neonatal outcomes is essential to safeguard the health of both pregnant women and fetus.

Mendelian randomization (MR) is an epidemiological approach that reveals causality in an unbiased manner, relying on genetic variation as the instrumental variable (IV) to assess whether an exposure leads to a corresponding outcome (Lawlor et al., 2008). Since alleles segregate according to Mendel’s second law of inheritance and genotypes are randomly assigned from parent to offspring without the influence of confounding, the causal sequence is reasonable (Burgess and Thompson, 2021). Given the infeasible conduction of randomized controlled trials, a number of MR studies have assessed the correlation between gut microbiota and pregnancy outcomes (Li C. et al., 2022; Li P. et al., 2022), while *H. pylori* was underexplored as the most common gastrointestinal pathogen.

In this study, we performed a two-sample MR analysis to evaluate the causal associations between *H. pylori* infection and nine pregnancy and neonatal outcomes, namely, miscarriage, preeclampsia or eclampsia, gestational diabetes mellitus, placental abruption, premature rupture of membranes, postpartum haemorrhage, birthweight, gestational age, and preterm birth.

## Methods and materials

### Study design

**Figure 1:**
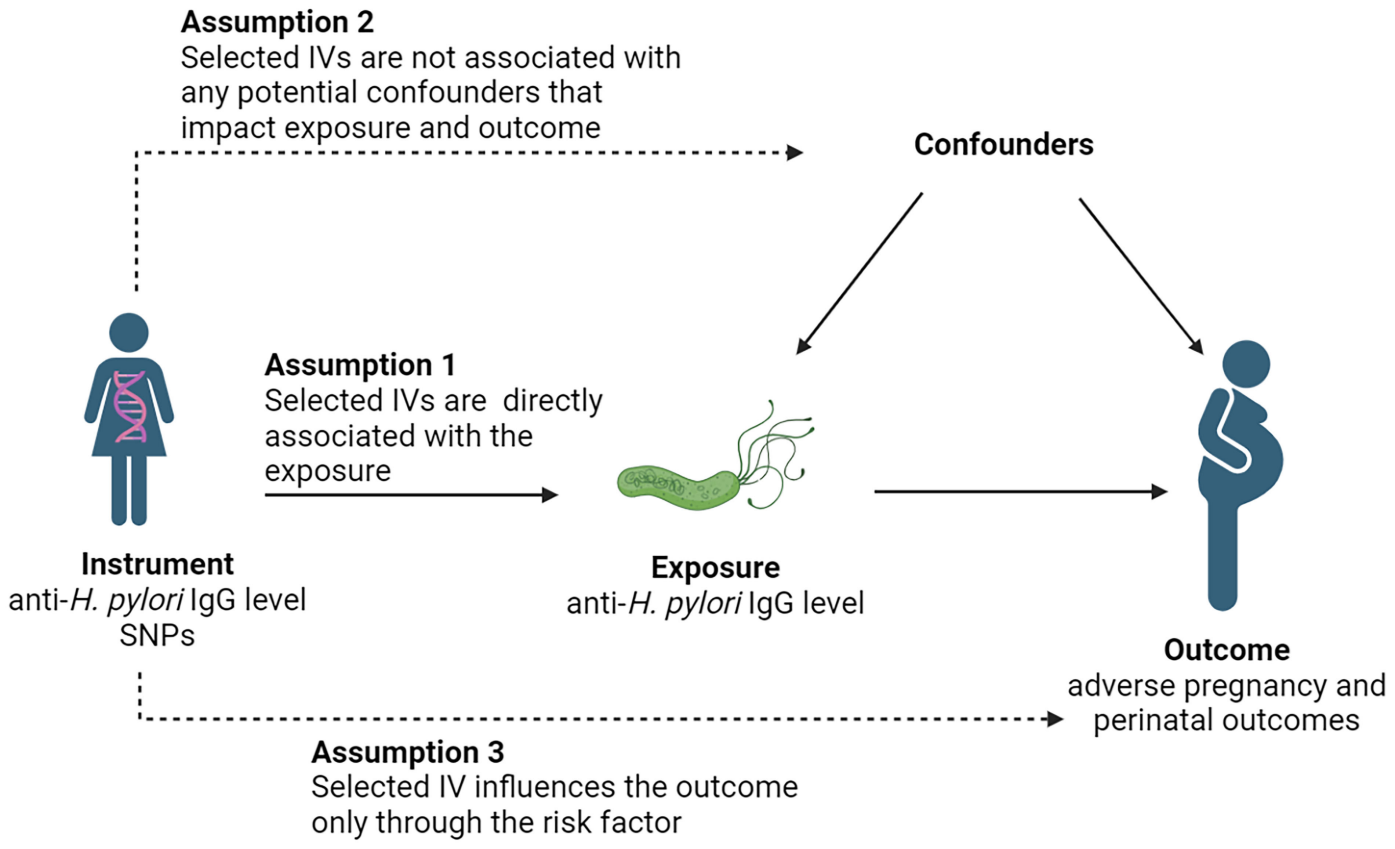
Study design. IV, instrumental variable; SNP, single nucleotide polymorphism.

A two-sample MR study was conducted using single nucleotide polymorphisms (SNPs) as IVs (Lawlor et al., 2008). *H. pylori* infection was defined on the basis of serum-specific IgG antibodies to *H. pylori*. Three core assumptions were used to ensure the accuracy of results (Davies et al., 2018): 1) each selected IV must be directly associated with the exposure; 2) each selected IV is not associated with any potential confounders that impact exposure and outcome; and 3) each selected IV influences the outcome only through the risk factor (**Figure 1**).

### Data sources

In this MR study, reliable genetic instruments were identified in large genome-wide association studies (GWAS). Exposure data were from the Avon Longitudinal Study of Parents and Children Cohort (ALSPAC), containing 4,735 individuals with anti-*H. pylori* IgG levels (Chong et al., 2021). Outcome data for miscarriage were obtained from GWAS meta-analysis by Laisk et al. (Laisk et al., 2020), including 49,996 sporadic cases and 174,109 controls. Other summary statistics for preeclampsia or eclampsia (Ncase = 3,903, Ncontrol = 114,735), gestational diabetes mellitus (Ncase = 5,687, Ncontrol = 117,892), placental abruption (Ncase = 294, Ncontrol = 104,247), premature rupture of membranes (Ncase = 3,011, Ncontrol = 104,247), postpartum haemorrhage (Ncase = 44,559, Ncontrol = 202,621), birthweight (N = 210,267), gestational age (N = 84,689), and preterm birth (Ncase = 4,775, Ncontrol = 60,148) were from FinnGen (https://www.finngen.fi/en) and Early Growth Genetics Consortium (www.egg-consortium.org) (Liu et al., 2019). All summary-level data used were harmonized and archived in the Medical Research Council Integrative Epidemiology Unit (MRC-IEU) OpenGWAS (https://gwas.mrcieu.ac.uk/). To reduce potential bias from population stratification, all SNPs and associated data were obtained from studies analyzed separately for those of European ancestry only. The characteristics of each GWAS dataset are detailed in **Supplementary Table 1**.

### IV selection

To ensure adequate screening for IVs, SNPs with genome-wide range significance levels less than the *P*-value (1×10^-5^) were selected (Sanna et al., 2019). Then, to check the independence of these variables and the effect of linkage disequilibrium, the SNP for the r^2^ was set to 0.001 and the clumping window size to 10,000 kb. In addition, IVs with F-statistics <10 were excluded to ensure the strength of association between IVs and exposure. The formula was F = r^2^×(N−1−K)/[(1−r^2^) ×K], where r^2^ represents the exposure variance explained by each IV, N denotes the sample size of the GWAS, and K refers to the number of instruments. Finally, we removed SNPs with minor allele frequency (MAF) less than the threshold of 0.01. Palindromic SNPs were also removed to ensure the effects of SNPs on exposure correspond to the same allele as the effects of SNPs on the outcome.

### MR analysis

A total of five methods, including inverse variance weighted (IVW), MR-Egger regression, weighted median, simple mode, and weighted mode, were used to evaluate whether there was a causal association between *H. pylori* infection and pregnancy and neonatal outcomes. In terms of algorithmic principles, the IVW method could integrate the Wald ratio for each SNP causal effect through meta-analysis. Without the horizontal pleiotropy, the IVW results would be unbiased (Bowden et al., 2017). The MR-Egger method can detect associations when the IV hypothesis does not apply but the weaker hypothesis does. It can also be used to assess horizontal pleiotropy and the results are consistent with IVW when an intercept term equals to zero, indicating the absence of horizontal pleiotropy (Bowden et al., 2015). The weighted median method can provide robust effect estimates when at least fifty percent of the instrumental information is valid, while the weighted mode is reliable if the largest subset of instruments with similar causal effects is valid (Hartwig et al., 2017).

### Heterogeneity and sensitivity analysis

Heterogeneity between IVs was analyzed using Cochran’s *Q*-test. Horizontal pleiotropy was assessed by the intercept of the MR-Egger method (Bowden et al., 2015) as well as the MR-pleiotropy residual sum and outlier (MR-PRESSO) global test (Verbanck et al., 2018). We also performed leave-one-out analyses to monitor whether causal associations were dominated by single SNPs, in which MR was performed iteratively to remove different SNPs using the “mr_leaveoneout_plot” program. All of the above analyses were performed in R version 4.2.1 (R Foundation for Statistical Computing, Vienna, Austria) and R packages TwoSampleMR (Hemani et al., 2017) and MR-PRESSO (Verbanck et al., 2018) were used.

## Results

According to the selection criteria of IVs, we identified a total of 20 SNPs for anti-*H. pylori* IgG levels. All F-statistics were above 10. The detailed information is shown in **Supplementary Table 2**.

Under the IVW method, genetically predicted IgG levels were significantly associated with increased risks of preeclampsia or eclampsia (odds ratio [OR] = 1.12, 95% confidence interval [CI] 1.01–1.24, *P* = 0.026) and premature rupture of membranes (OR = 1.17, 95% CI 1.05–1.30, *P* = 0.004). For preeclampsia or eclampsia, similar results were obtained via MR-Egger (OR = 1.32, 95% CI 1.06–1.64, *P* = 0.027). For premature rupture of membranes, the OR estimates obtained from the weighted median (OR = 1.22, 95% CI 1.06–1.41, *P* = 0.006) were also consistent with those from IVW. No significant associations were observed in sporadic miscarriage, gestational diabetes mellitus, placental abruption, postpartum haemorrhage, birthweight, gestational age, and preterm birth. The results of the five MR analysis methods are displayed in **Table 1**.

**Table 1:** MR estimates for the association between *H. pylori* infection and nine pregnancy and neonatal outcomes.

Outcome	MR method	No. of SNP	OR	95% CI	*P*-value
Miscarriage	IVW	20	1.01	0.97–1.04	0.712
	MR-Egger	20	1.10	1.03–1.18	0.009
	Simple mode	20	1.05	0.96–1.15	0.304
	Weighted median	20	1.03	0.99–1.08	0.122
	Weighted mode	20	1.05	0.96–1.15	0.288
Preeclampsia or eclampsia	IVW	18	1.12	1.01–1.24	0.026
	MR-Egger	18	1.32	1.06–1.64	0.027
	Simple mode	18	1.21	0.94–1.57	0.161
	Weighted median	18	1.13	0.98–1.30	0.101
	Weighted mode	18	1.25	0.99–1.57	0.074
Gestational diabetes mellitus	IVW	20	0.98	0.90–1.06	0.568
	MR-Egger	20	0.98	0.82–1.17	0.803
	Simple mode	20	1.01	0.84–1.22	0.903
	Weighted median	20	0.99	0.88–1.11	0.845
	Weighted mode	20	1.00	0.83–1.22	0.963
Placental abruption	IVW	20	1.18	0.82–1.70	0.371
	MR-Egger	20	1.67	0.74–3.80	0.234
	Simple mode	20	0.94	0.41–2.14	0.877
	Weighted median	20	0.97	0.59–1.58	0.891
	Weighted mode	20	0.88	0.41–1.89	0.755
Premature rupture of membranes	IVW	20	1.17	1.05–1.30	0.004
	MR-Egger	20	1.13	0.89–1.43	0.331
	Simple mode	20	1.27	0.99–1.64	0.075
	Weighted median	20	1.22	1.06–1.41	0.006
	Weighted mode	20	1.25	0.99–1.58	0.076
Postpartum hemorrhage	IVW	13	1.00	0.91–1.10	0.947
	MR-Egger	13	0.85	0.65–1.11	0.256
	Simple mode	13	0.97	0.83–1.13	0.669
	Weighted median	13	1.00	0.91–1.11	0.939
	Weighted mode	13	0.99	0.87–1.12	0.816
Birthweight	IVW	19	0.99	0.98–1.01	0.398
	MR-Egger	19	1.01	0.97–1.05	0.673
	Simple mode	19	0.97	0.93–1.01	0.194
	Weighted median	19	0.99	0.97–1.01	0.156
	Weighted mode	19	0.97	0.94–1.01	0.171
Gestational age	IVW	18	0.99	0.97–1.02	0.549
	MR-Egger	18	0.95	0.91–1.00	0.062
	Simple mode	18	0.98	0.93–1.03	0.464
	Weighted median	18	0.98	0.96–1.01	0.294
	Weighted mode	18	0.98	0.93–1.03	0.404
Preterm birth	IVW	18	1.05	0.95–1.16	0.366
	MR-Egger	18	1.13	0.90–1.42	0.313
	Simple mode	18	1.16	0.89–1.51	0.278
	Weighted median	18	1.12	0.97–1.29	0.137
	Weighted mode	18	1.15	0.88–1.50	0.311

MR, mendelian randomization; IVW, inverse variance weighted; SNP, single nucleotide polymorphism; OR, odds ratio; CI, confidence interval.

**Figure 2:**
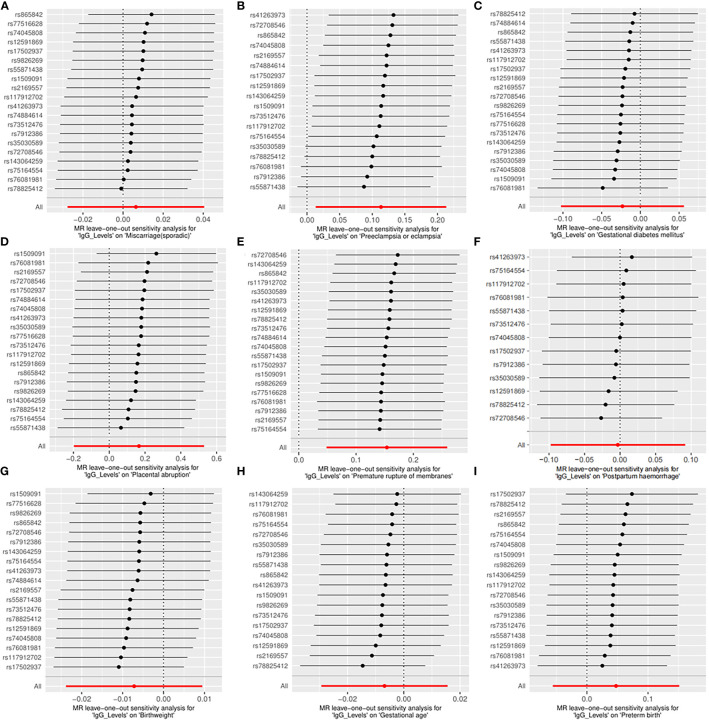
Leave-one-out plots for the causal associations between anti-*H. pylori* IgG levels and nine pregnancy and neonatal outcomes. **(A)** Sporadic miscarriage. **(B)** Preeclampsia or eclampsia. **(C)** Gestational diabetes mellitus. **(D)** Placental abruption. **(E)** Premature rupture of membranes. **(F)** Postpartum haemorrhage. **(G)** Birthweight. **(H)** Gestational age. **(I)** Preterm birth. MR, mendelian randomization.

**Figure 3:**
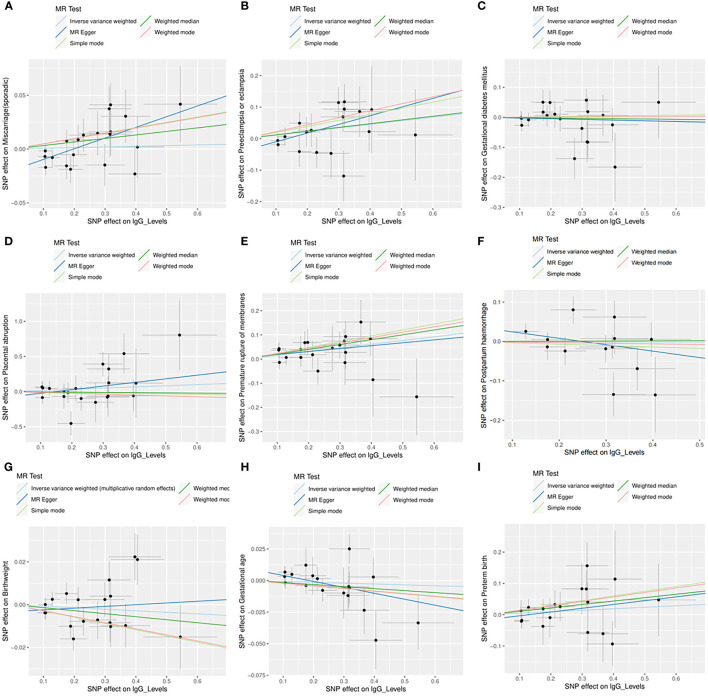
Scatter plots for the causal associations between anti-*H. pylori* IgG levels and nine pregnancy and neonatal outcomes. **(A)** Sporadic miscarriage. **(B)** Preeclampsia or eclampsia. **(C)** Gestational diabetes mellitus. **(D)** Placental abruption. **(E)** Premature rupture of membranes. **(F)** Postpartum haemorrhage. **(G)** Birthweight. **(H)** Gestational age. **(I)** Preterm birth. Five MR methods are indicated by different colors, including inverse variance weighted (light blue), MR Egger (dark blue), weighed median (dark green), weighted mode (red), and simple mode (light green). SNP, single nucleotide polymorphism; MR, mendelian randomization.

Across the MR study, no evidence of directional pleiotropy and heterogeneity was found in the MR-PRESSO global test, MR-Egger intercept test, and Cochran’s IVW and MR-Egger *Q* tests, except for analyzing the causal relationship between *H. pylori* infection and birthweight (**Table 2**). The results of the leave-one-out permutation analysis showed that the overall risk estimate was not driven by certain SNPs (**Figure 2**). In addition, potential outliers and the effects of SNPs, individually and jointly, from each MR method were shown in scatter plots (**Figure 3**), which displayed a similar trend toward a positive MR association of anti-*H. pylori* IgG levels with preeclampsia or eclampsia and premature rupture of membranes.

**Table 2:** Pleiotropy and heterogeneity tests of IVs for nine pregnancy and neonatal outcomes.

Outcome	Pleiotropy		Heterogeneity	
	MR-PRESSO (*P*-value)	MR-Egger (*P*-value)	MR Egger (*P*-value)	IVW (*P*-value)
Miscarriage	0.168	0.007	0.589	0.152
Preeclampsia or eclampsia	0.414	0.129	0.496	0.391
Gestational diabetes mellitus	0.494	0.999	0.423	0.489
Placental abruption	0.210	0.364	0.214	0.216
Premature rupture of membranes	0.865	0.761	0.821	0.859
Postpartum hemorrhage	0.085	0.237	0.082	0.058
Birthweight	0.041	0.373	0.037	0.036
Gestational age	0.309	0.070	0.518	0.337
Preterm birth	0.595	0.489	0.602	0.635

IV, instrumental variable; MR, mendelian randomization; PRESSO, pleiotropy residual sum and outlier; IVW, inverse variance weighted.

## Discussion

In this MR study, we found that anti-*H. pylori* IgG level was causally associated with preeclampsia or eclampsia and premature rupture of membranes, but not associated with other pregnancy (miscarriage, gestational diabetes mellitus, placental abruption, and postpartum haemorrhage) and neonatal (birthweight, gestational age, and preterm birth) outcomes.

Emerging evidence has shown the role of *H. pylori* infection in preeclampsia-eclampsia. In 2006, Ponzetto et al. (Ponzetto et al., 2006) first reported that pregnant women with preeclampsia had a 19.2% higher rate of *H. pylori* seropositivity compared with uncomplicated women. Following that, other groups also demonstrated an epidemiological association between *H. pylori* infection and preeclampsia, especially for women who were infected with cytotoxin-associated gene A (CagA) positive strains (Mosbah and Nabiel, 2016; Bellos et al., 2018; Nourollahpour Shiadeh et al., 2019). *In vitro* studies further showed that anti-CagA antibodies could cross-react with cytotrophoblast cells through β-actin, thus reducing their invasiveness by decreasing ERK 1/2 activation, NF-kB translocation and MMP-2 expression (Franceschi et al., 2012). Since trophoblast invasion of maternal decidua is vital for embryo implantation and placental development, the infection-induced autoimmunity may lead to inadequate placentation and preeclampsia onset (Tersigni et al., 2014). In addition, Di Simone et al. (Di Simone et al., 2017) found that *H. pylori* infection was associated with abnormality of uterine arteries Doppler velocimetry in preeclamptic women, and anti-*H. pylori* IgG fractions from these women could inhibit endothelial cells’ proliferation, migration and differentiation both *in vitro* and *in vivo*. Therefore, *H. pylori* infection may also have an impact on the angiogenesis and vascular resistance, which constitutes an important mechanism of preeclampsia pathogenesis (Chaiworapongsa et al., 2014).

For the correlation with premature rupture of membranes, the mechanisms are still unclear but may be associated with systematic and local effects of *H. pylori* infection. On the one hand, *H. pylori* could stimulate the release of pro-inflammatory cytokines, such as interleukin (IL)-1β, IL-6, IL-8, tumor necrosis factor-α, and macrophage migration inhibitory factor (Xia et al., 2005; UstUn et al., 2010; Chen et al., 2022). Among infected patients, systemic indices of inflammation were also observed to be elevated, including white blood cell count and C-reactive protein (Graham et al., 1998; Oshima et al., 2005; UstUn et al., 2010). Given the crucial role of the inflammation-oxidative stress axis in fetal membrane weakening (Menon and Richardson, 2017), the infection may thus lead to premature rupture of membranes. On the other hand, research found that extracellular vesicles (EVs) could be derived from *H. pylori*-infected gastric epithelial cells and entered the blood circulation (Xia et al., 2020). Additionally, outer membrane vesicles (OMVs) released by *H. pylori* also existed in the serum samples (Park and Tsunoda, 2022). Both EVs and OMVs could serve as transport vehicles to deliver pathogenic virulence factors (e.g., CagA) to extragastric organs including brain (Qiang et al., 2022; Xie et al., 2023). In this regard, the fetal membrane may be directly affected as well, while further studies are warranted for investigation.

Consistent with previous pooled results (Tang et al., 2021), our study did not support the link between *H. pylori* infection and preterm birth. However, several cohorts have shown an increased risk of miscarriage (Hajishafiha et al., 2011), gestational diabetes mellitus (Cardaropoli et al., 2015; Li et al., 2020; Tang et al., 2021), and low birth weight (Wanyama et al., 2016; Grooten et al., 2017; Zhan et al., 2019) among infected pregnant women, which were not detected by the current MR analysis. This may be due to the insufficiency of cases as well as residual confounding of observational design. As for placental abruption and postpartum haemorrhage, no relevant research is available thus far and their associations with *H. pylori* infection remain to be explored.

To our knowledge, this is the first MR study on the association of *H. pylori* infection with a series of pregnancy and neonatal outcomes, thus eliminating the interference of confounding factors and reverse causation. To avoid sample overlap and its associated bias, we used the exposure and outcome datasets from multiple independent GWAS. An iterative MR analysis with five different approaches was conducted to acquire conservative results, and the absence of pleiotropy and heterogeneity in most sensitivity analyses further rule out the false-positive likelihood.

There are some limitations of the current study that should be acknowledged. Firstly, in order to minimize demographic bias, we selected only data from people of European descent, making the generalizability of our finding to other ethnic populations compromised. Secondly, the GWAS sample size of *H. pylori* infection was relatively small. Based on the traditional GWAS significance threshold (*P <*5×10^−8^), the SNPs obtained were too few for further study. Therefore, we used the locus-wide significance level (*P <*1×10^-5^) for SNP selection, which may introduce weak instrument bias to the overall estimates. Thirdly, our analysis was based on summary statistics instead of raw data, and it was not possible to conduct further subgroup analyses on the specific strain of *H. pylori* (e.g., CagA-positive and CagA-negative) and severe degree or subtype of disease (e.g., early-onset and late-onset preeclampsia). Lastly, serological tests measuring the overall antibody immune response towards *H. pylori* are incapable of distinguishing ongoing from past infection. For a more specific differentiation, additional information is needed such as ^13^C-urea breath test, histological examination, or eradication record (Sabbagh et al., 2019). Therefore, the present study using genetically-predicted *H. pylori* seropositivity only represent the susceptibility to infection, and further studies are needed to compare the outcomes of different infection status.

## Conclusion

In summary, this two-sample MR study showed that genetically predicted *H. pylori* infection was causally associated with increased risks of preeclampsia-eclampsia and premature rupture of membranes. Our findings confirm the epidemiological evidence on the adverse impact of *H. pylori* in pregnancy. Further studies are needed to thoroughly elucidate the pathophysiological mechanisms and assess the effectiveness of pre-pregnancy screening and preventive eradication.

## Data availability statement

All GWAS summary data used are publicly available. The download links are detailed in **Supplementary Table 1**.

## Ethics statement

Ethical approval was not required because this study was based solely on publicly available GWAS summary-level statistics and no individual-level data were used.

## Author contributions

JH: Formal analysis, Funding acquisition, Writing – original draft. YL: Formal analysis, Writing – original draft. DX: Investigation, Writing – review & editing. MC: Data curation, Writing – review & editing. QX: Data curation, Writing – review & editing. JC: Data curation, Writing – review & editing. LX: Data curation, Writing – review & editing. LY: Data curation, Writing – review & editing. QW: Data curation, Writing – review & editing. ZL: Conceptualization, Project administration, Writing – review & editing. JW: Conceptualization, Supervision, Writing – review & editing. LT: Conceptualization, Supervision, Writing – review & editing.
