# Supplementary material for: Causal associations between Helicobacter pylori infection and pregnancy and neonatal outcomes: a two-sample Mendelian randomization study
Source: Front Cell Infect Microbiol. 2024 Mar 14;14:1343499. doi: 10.3389/fcimb.2024.1343499 (PMC10979540; doi:10.3389/fcimb.2024.1343499)
Supplement: Supplementary file 1 [file Table_1.docx]

**Table S1.** Genome-wide association analyses used in the study.

| **Phenotype** | **Source** | **Sample size (N)** | | | **Links** |
| --- | --- | --- | --- | --- | --- |
|  |  | **Cases** | **Controls** | **Total** |  |
| Anti-*H. pylori*  IgG levels | Chong *et al.* | – | – | 4,683 | https://gwas.mrcieu.ac.uk/datasets/ieu-b-4905/ |
| Miscarriage | Laisk *et al.* | 49,996 | 174,109 | 224,105 | https://www.ebi.ac.uk/gwas/studies/GCST011888 |
| Preeclampsia or eclampsia | FinnGen | 3,903 | 114,735 | 118,638 | https://gwas.mrcieu.ac.uk/datasets/finn-b-O15_PRE_OR_ECLAMPSIA/ |
| Gestational diabetes mellitus | FinnGen | 5,687 | 117,892 | 123,579 | https://gwas.mrcieu.ac.uk/datasets/finn-b-GEST_DIABETES/ |
| Placental abruption | FinnGen | 294 | 104,247 | 104,541 | https://gwas.mrcieu.ac.uk/datasets/finn-b-O15_PLAC_PREMAT_SEPAR/ |
| Premature rupture of membranes | FinnGen | 3,011 | 104,247 | 107,258 | https://gwas.mrcieu.ac.uk/datasets/finn-b-O15_MEMBR_PREMAT_RUPT/ |
| Postpartum hemorrhage | FinnGen | 8,249 | 202,621 | 210,870 | https://r9.finngen.fi/pheno/O15_POSTPART_HEAMORRH/ |
| Birthweight | EGG  Consortium | – | – | 210,267 | http://egg-consortium.org/birth-weight-2019.html |
| Gestational age | EGG  Consortium | – | – | 84,689 | http://egg-consortium.org/gestational-duration-2019.html |
| Preterm birth | EGG  Consortium | 4,775 | 60,148 | 64,923 | http://egg-consortium.org/gestational-duration-2019.html |

EGG, Early Growth Genetics.
